# Reliability of an Assessment Method for Resilience

**DOI:** 10.1192/j.eurpsy.2025.1608

**Published:** 2025-08-26

**Authors:** L. Mehl-Madrona, B. Mainguy

**Affiliations:** 1 Native Studies, University of Maine, Orono; 2 Psychiatry Residency, Northern Light Acadia; 3 Wabanaki Health and Wellness, Bangor, United States

## Abstract

**Introduction:**

Understanding resilience becomes important given that adversity is an unavoidable fact of human life. Over 70% of respondents in a sample of 68,894 people reported at least one traumatic event in their lifetime. The neurotoxic effects of these experiences range from compromised neurocompetence, psychopathology including PTSD and depression, to adverse physical effects.

**Objectives:**

To determine if we could reliably agree on ratings of resilience on a five point scale to aid future studies of the role of resilience in recovery and relapse.

**Methods:**

To assess resilience we developed a five point scale with 1 being the least and 5 behind the most resilient pattern. We found a water metaphor useful to conceptualize these five levels of resilience. Resilience in an ever-changing world can be likened to navigating in a body of water. Adversity tosses us into the water. Here are our levels:

**Level 1:** At this level, the person sinks to the bottom and remains there. They are making no effort to change their circumstances and remain stagnant.

**Level 2:** At this level, the person is not sitting at the bottom, but has not yet reached the surface. They are in a place of struggle and resistance, where change is elusive.

**Level 3:** The person is treading water at this stage. They are working hard to stay afloat, but not making significant progress in altering their overall situation.

**Level 4:** They’re swimming toward shore, toward a more favorable environment in which they can thrive. They’re actively seeking change and adjustment to a post-adversity reality.

**Level 5:** At this point, they’ve managed to climb out of the water and change their circumstances. Their resilience allows them to overcome challenges and seek better surroundings.

We used kappa statistics to assess our level of agreement among ourselves (three raters) after studying and discussing prototypical stories for each rating level.

**Results:**

Practicing with training videos, we found we could achieve 84% agreement on the five ratings with 3 raters.

Percent overall agreement = 84.00% Free-marginal kappa = 0.80

95% CI for free-marginal kappa [0.66, 0.94]; Fixed-marginal kappa = 0.78; 95% CI for fixed-marginal kappa [0.62, 0.93]

When we added AI, we got Percent overall agreement = 71.33% Free-marginal kappa = 0.64; 95% CI for free-marginal kappa [0.50, 0.79]

Fixed-marginal kappa = 0.61; 95% CI for fixed-marginal kappa [0.51, 0.71]

**Conclusions:**

The individuals who fit into the pattern of low resilience tended to have a high amount of childhood adverse experiences as shown through the ACE survey. The accumulation of these events in combination with external variables shape resilience. Factors including intelligence/education level, drug/alcohol use, positive role models, exposures to nature/art/spirituality, and community/family norms steer a person down a set of patterned thinking and actions which ultimately depict their overall life story.

**Disclosure of Interest:**

None Declared

